# A national survey of state laws regarding medications for opioid use disorder in problem-solving courts

**DOI:** 10.1186/s40352-022-00178-6

**Published:** 2022-03-31

**Authors:** Barbara Andraka-Christou, Olivia Randall-Kosich, Matthew Golan, Rachel Totaram, Brendan Saloner, Adam J. Gordon, Bradley D. Stein

**Affiliations:** 1 School of Global Health Management & Informatics, University of Central Florida, 500 W Livingston Street, Orlando, FL 32801 USA; 2 Department of Internal Medicine (Secondary Joint Appointment), University of Central Florida, 500 W Livingston Street, Orlando, FL 32801 USA; 3 School of Public Health, Georgia State University, Atlanta, GA USA; 4 School of Law, Emory University, Atlanta, GA USA; 5 Bloomberg School of Public Health, Johns Hopkins University, Baltimore, MD USA; 6 Informatics, Decision-Enhancement, and Analytic Sciences (IDEAS) Center, VA Salt Lake City Health Care System, Salt Lake City, UT USA; 7 Program for Addiction Research, Clinical Care, Knowledge and Advocacy (PARCKA), Division of Epidemiology, Department of Internal Medicine, University of Utah School of Medicine, Salt Lake City, UT USA; 8 RAND Corporation, Pittsburgh, PA USA

**Keywords:** Problem-solving courts, Methadone, Buprenorphine, Drug court, Medication for opioid use disorder, State law

## Abstract

**Background:**

Problem-solving courts have the potential to help reduce harms associated with the opioid crisis. However, problem-solving courts vary in their policies toward medications for opioid use disorder (MOUD), with some courts discouraging or even prohibiting MOUD use. State laws may influence court policies regarding MOUD; thus, we aimed to identify and describe state laws related to MOUD in problem-solving courts across the US from 2005 to 2019.

**Methods:**

We searched Westlaw legal software for regulations and statutes (collectively referred to as “state laws”) in all US states and D.C. from 2005 to 2019 and included laws related to both MOUD and problem-solving courts in our analytic sample. We conducted a modified iterative categorization process to identify and analyze categories of laws related to MOUD access in problem-solving courts.

**Results:**

Since 2005, nine states had laws regarding MOUD in problem-solving courts. We identified two overarching categories of state laws: 1) laws that prohibit MOUD bans, and 2) laws potentially facilitating access to MOUD. Seven states had laws that prohibit MOUD bans, such as laws prohibiting exclusion of participants from programs due to MOUD use or limiting the type of MOUD, dose or treatment duration. Four states had laws that could facilitate access to MOUD, such as requiring courts to make MOUD available to participants.

**Discussion:**

Relatively few states have laws facilitating MOUD access and/or preventing MOUD bans in problem-solving courts. To help facilitate MOUD access for court participants across the US, model state legislation should be created. Additionally, future research should explore potential effects of state laws on MOUD access and health outcomes for court participants.

**Supplementary Information:**

The online version contains supplementary material available at 10.1186/s40352-022-00178-6.

## Introduction

Problem-solving courts are an alternative to traditional courts, and commonly involve mandated treatment and wrap-around services (e.g., connection to employment resources, peer support specialists) to facilitate recovery for justice-involved people with substance use disorder (SUD) (Center for Children and Family Futures and National Association of Drug Court Professionals, 2019; Marlowe et al., 2016; National Association of Drug Court Professionals, 2015). These courts typically have interdisciplinary teams and are led by a judge. Problem-solving courts, including adult drug courts, veterans’ courts, mental health courts, and family dependency courts, help participants with SUD avoid a criminal charge, or incarceration related to drug activity or reunify with children whose custody they have lost due to drug use (Center for Children and Family Futures and National Association of Drug Court Professionals, 2019; Sieger & Haswell, 2020).

Problem-solving courts are receiving growing interest as a potential way to mitigate harms related to the opioid crisis (National Academies of Science Engineering & Medicine, 2019), with increasing attention regarding whether problem-solving courts facilitate access to medications for opioid use disorder (MOUD). MOUDs—methadone, buprenorphine, and naltrexone—are the gold standard treatment for OUD and improve patient outcomes (Jarvis et al., 2018; Nielsen et al., 2016). However, some problem-solving courts appear to prohibit MOUD use (Matusow et al., 2013) or require MOUD cessation prior to court program completion (Andraka-Christou et al., 2021a). Court policies are more likely to disfavor methadone and buprenorphine than naltrexone (Matusow et al., 2013; Andraka-Christou et al., 2022; Andraka-Christou, 2017), reflecting more negative beliefs about agonist MOUD among many court team members (Matusow et al., 2013; Andraka-Christou, 2017; Andraka-Christou & Atkins, 2020a; Andraka-Christou et al., 2019). A recent study using a convenience sample found that 90% of Florida problem-solving courts are deciding whether to permit participant MOUD use on a case-by-case basis, but the factors used by these courts in their decision-making process are unknown (Andraka-Christou et al., 2022). Negative attitudes toward MOUD among problem-solving court team members may also contribute to low referral rates of people with OUD from problem-solving courts to methadone and buprenorphine treatment (Krawczyk et al., 2020).

Problem-solving courts typically operate autonomously. For example, two adult problem-solving courts in the same county can have very different MOUD policies and practices. Additionally, courts may have different policies toward different MOUDs, particularly since extended-release naltrexone became available in the US in 2011 and is not a controlled substance. In 2013 and 2015, following a study finding widespread prohibition of agonist MOUD in adult drug courts (Matusow et al., 2013), the National Association of Drug Court Professionals (NADCP), a non-governmental professional body, released voluntary best practice standards that would require problem-solving courts to allow MOUD use (National Association of Drug Court Professionals, 2015). Also, as of 2015 the federal government has required federally funded problem-solving courts to allow MOUD (Davies, 2015), but hundreds of problem-solving courts do not receive federal funding, relying instead on state and/or local funding. Therefore, it is critical to examine state laws relating to MOUD in problem-solving courts, which could promote or discourage MOUD even when a court does not receive federal funding or does not voluntarily follow NADCP standards. We are unaware of a review of state laws related to MOUD use and problem-solving courts. To address this gap in the literature, this paper aims to identify and describe the types of state laws related to MOUD in drug courts across the US enacted between 2005 and 2019, including identifying the states that have each type of law.

## Methods

To identify state laws regarding MOUD access for participants in problem-solving courts, we systematically reviewed and analyzed relevant statutes and regulations (hereafter, collectively defined as state laws) in all US states and the District of Columbia from 2005 to 2019.

### Data collection

This research is part of a larger project collecting and analyzing state laws related to MOUD (Andraka-Christou et al., 2021b). We searched Westlaw legal software to identify relevant laws across 51 jurisdictions (all states and Washington, D.C.) in effect at any time between 2005 and 2019, as laws prior to 2005 were not consistently available in Westlaw. We identified laws that included terms related to MOUD, such as buprenorphine, methadone, and naltrexone; however, search terms did not include brand names (e.g., Vivitrol, Suboxone; see Appendix A for detailed search terms). We subsequently excluded laws unrelated to SUD treatment, such as those only related to pain management. Our search resulted in over 5000 state laws related to SUD treatment across 51 jurisdictions, with details regarding the data collection process reported elsewhere (Andraka-Christou et al., 2021b). As is typical in legal epidemiological research, each Westlaw search result was considered a separate law for purposes of analysis, even if some results were related to the same title or section of a statute/regulation (Ibrahim et al., 2011; Burris, 2021). Results were uploaded into Dedoose software for further analysis (Dedoose Version 8.0.35, 2018).

For the current analysis, the research team used Dedoose software search features to identify laws applicable to problem-solving courts. We identified such laws by using the software’s search feature to find laws including any of the following terms: “diversion program,” “diversionary”, “court,” or “criminal”; and we then examined the text of the law for applicability to problem-solving courts. If a law applied to diversion programs (i.e., programs designed to divert individuals away from incarceration) without specifying problem-solving courts, we nevertheless included the law, as problem-solving courts are a type of diversionary program. Since our focus was on laws preventing or requiring actions of actors in the court system, we excluded laws only applicable to actors outside of the court system. For example, we excluded laws requiring opioid treatment programs to document patients’ involvement in courts. A total of 37 laws were in our final analytical sample. See Appendix B for a PRISMA flow diagram.

### Data analysis

To identify categories of laws related to MOUD access in problem-solving courts, four research team members (BA, ORK, MG, RT) conducted a modified iterative categorization process (Neale, 2016) of the final data sample. The four team members included a J.D./Ph.D. health services and policy researcher (BA), two Ph.D. student researchers (ORK and RT), and one law student researcher (MG), all of whom have significant experience conducting qualitative analysis of policies related to MOUD. First, we created a preliminary codebook based on a review of the 37 laws in the final sample, which included the following codes: “can require MOUD of the participant,” “must assess for MOUD or connect to MOUD treatment,” “can allow MOUD, but is not required to assess for MOUD or connect to MOUD treatment,” “cannot prohibit MOUD treatment,” “cannot require special type of medication (e.g., xr-naltrexone),” “pilot programs,” and “adjunctive psychosocial services requirements.” Next, the four team members independently coded each law. Then, the researchers met regularly in teams of two (BA/RT and ORK/MG) to review findings, discuss discrepancies in independent coding, and identify any needed changes to the codebook. After iterative revisions, codes were grouped into the following three categories: “MOUD prohibition,” “MOUD facilitation,” and “other MOUD laws.”

Next, excerpts for each of these three categories were exported into Excel spreadsheets and two team members independently created a label/summary for each excerpt, with any discrepancies in labeling resolved through discussion. For example, one excerpt in the MOUD prohibition category was labeled “MOUD use cannot be considered a reason for unsuccessful completion of the court program.” All labels were examined by an attorney and Ph.D.-level health services and policy researcher with subject matter expertise in MOUD, who also reviewed and subsequently categorized any uncategorized labels. During this process, after team discussion we decided that laws in the “other” category, which allowed courts to *require* participants to use MOUD, were regrouped into the facilitation category. A domain summary was created for each category, with representative examples, which was then discussed with the larger team. The number of states with at least one law in each category and the effective years of each law were also examined.

## Results

Nine states (including Washington D.C.) had a total of 37 state laws regarding MOUD in problem-solving courts or diversion programs: California, Washington D.C., Illinois, Indiana, Mississippi, Missouri, New Jersey, New York, and Washington. Of the 37 laws, most were recent, with only two laws effective prior to 2015. We identified two overarching categories of state laws, described in greater detail below: laws that prohibit MOUD bans in problem-solving courts (*n* = 24; 64%) and laws potentially facilitating access to MOUD in problem-solving courts (*n* = 13; 36%).

### Laws that prohibit MOUD bans

Seven states (California, Washington D.C., Illinois, Missouri, New Jersey, New York, and Washington) had 24 laws prohibiting MOUD bans in problem-solving courts or, more broadly, diversionary programs. These laws prohibited one or more of the following: exclusion of participants from programs due to MOUD use; considering MOUD use a violation of program rules or prohibiting MOUD during the program; requiring participants to reduce or stop MOUD for successful *completion* of the program; or limiting the type of MOUD, dose or treatment duration. Some states, like Missouri, explicitly prohibit courts from favoring one type of MOUD, while other states imply all types of MOUD must be permitted. In some cases, laws prohibiting MOUD bans do not reference healthcare providers, while other laws apply when a licensed healthcare provider (or “a licensed physician” in Illinois and Missouri) recommends or prescribes MOUD. See Table 1 for the relevant legal text and effective years of laws related to MOUD bans.

**Table 1:** Laws prohibiting MOUD bans

State	Effective Years	Type of action prohibited	Applicability	Relevant legal text (citation)
CA	2018-2019	Cannot exclude from program because of MOUD use	Pretrial or pre-guilty plea diversion program	[T]he use by a participant of medications to treat substance use disorders shall not be the sole reason for exclusion from a pretrial diversion or preguilty plea program (§ 1000.6) (Cal. Penal Code § 1000.6 (West))
	2018-2019	Cannot consider MOUD a violation of program rules	Pretrial or pre-guilty plea diversion program	Urinalysis results that only establish that a person described in this section has ingested medication duly prescribed to that person by his or her physician or psychiatrist, or medications used to treat substance use disorders, shall not be considered a violation of the terms of the pretrial diversion or preguilty plea program under this chapter (Cal. Penal Code § 1000.6 (West))
	2018-2019	Cannot prohibit MOUD; cannot restrict type of MOUD *(implied)*	Pretrial or pre-guilty plea diversion program	A person who is participating in a pretrial diversion program or a preguilty plea program pursuant to this chapter is authorized under the direction of a licensed health care practitioner, to use medications including, but not limited to, methadone, buprenorphine, or levoalphacetylmethadol (LAAM) to treat substance use disorders if the participant allows release of his or her medical records to the court presiding over the participant's preguilty plea or pretrial diversion program for the limited purpose of determining whether or not the participant is using such medications under the direction of a licensed health care practitioner and is in compliance with the pretrial diversion or preguilty plea program rules. (Cal. Penal Code § 1000.6 (West))
DC	2005-2006	Cannot require reducing or stopping MOUD for successful program completion; cannot restrict type of MOUD *(implied)*	Pre or post trial diversion program	Successful completion of treatment shall not require the reduction or cessation of narcotic replacement therapies, including methadone maintenance treatment. (D.C. Code Ann. § 24-751.10 (West 2003)
IL	2017-2019	Cannot consider MOUD a violation of program rules	Criminal drug court	A defendant who is assigned to a substance abuse treatment program under this Act for opioid abuse or dependence is not in violation of the terms or conditions of the program on the basis of his or her participation in medication assisted treatment under the care of a physician licensed in this State to practice medicine in all of its branches. (730 Ill. Comp. Stat. Ann. 166/35 (West))
		Cannot prohibit MOUD use; cannot require reducing or stopping MOUD for successful program completion	Criminal drug court	If the defendant needs treatment for opioid abuse or dependence, the court may not prohibit the defendant from participating in and receiving medication assisted treatment under the care of a physician licensed in this State to practice medicine in all of its branches. Drug court participants may not be required to refrain from using medication assisted treatment as a term or condition of successful completion of the drug court program. (730 Ill. Comp. Stat. Ann. 166/25 (West))
MO	2018-2019	Cannot prohibit using MOUD; Cannot require reducing or stopping MOUD for successful program completion; Cannot consider MOUD a violation of program rules	Family court	If a family court participant requires treatment for opioid or other substance misuse or dependence, a family court shall not prohibit such participant from participating in and receiving medication-assisted treatment under the care of a physician licensed in this state to practice medicine. A family court participant shall not be required to refrain from using medication-assisted treatment as a term or condition of successful completion of the family court program. 3. A family court participant assigned to a treatment program for opioid or other substance misuse or dependence shall not be in violation of the terms or conditions of the family court on the basis of his or her participation in medication-assisted treatment under the care of a physician licensed in this state to practice medicine. (Mo. Ann. Stat. § 487.200 (West))
	2019	Cannot prohibit using MOUD; Cannot require reducing or stopping MOUD for successful program completion; Cannot consider MOUD a violation of program rules	Treatment courts	If a treatment court participant requires treatment for opioid or other substance misuse or dependence, a treatment court shall not prohibit such participant from participating in and receiving medication-assisted treatment under the care of a physician licensed in this state to practice medicine. A treatment court participant shall not be required to refrain from using medication-assisted treatment as a term or condition of successful completion of the treatment court program. 5. A treatment court participant assigned to a treatment program for opioid or other substance misuse or dependence shall not be in violation of the terms or conditions of the treatment court on the basis of his or her participation in medication-assisted treatment under the care of a physician licensed in this state to practice medicine. (Mo. Ann. Stat. § 478.004 (West))
	2019	Cannot restrict type of MOUD, dose, or duration	Drug courts and other diversion programs	The court or other diversion program …. shall not impose any limitations on the type of medication or other treatment prescribed or the dose or duration of MAT recommended by the physician. (Mo. Ann. Stat. § 191.1165 (West))
NJ	2015-2019	Cannot require reducing or stopping MOUD for successful program completion; Cannot consider MOUD a violation of program rules; cannot restrict type of MOUD	Special probation (i.e., diversion programs)	In the case of the temporary or continued management of a person's drug or alcohol dependency by means of medication-assisted treatment as defined herein, whenever supported by a report from the treatment provider of existing satisfactory progress and reasonably predictable long-term success with or without further medication-assisted treatment, the person's use of the medication-assisted treatment, even if continuing, shall not be the basis to constitute a failure to complete successfully the treatment program. 2C:35-14(e) If the person is involved with a program that is providing the person medication-assisted treatment as defined in paragraph (7) of subsection f. of this section, only a positive urine test for drug or alcohol use unrelated to the medication-assisted treatment shall constitute a violation of the terms and conditions of special probation. (2C:35-14(f)(7)) As used in this section, the term “medication-assisted treatment” means the use of any medications approved by the federal Food and Drug Administration to treat substance use disorders, including extended-release naltrexone, methadone, and buprenorphine, in combination with counseling and behavioral therapies, to provide a whole-patient approach to the treatment of substance use disorders. (2C:35-14(f)(7)) 2015 -> (N.J. Stat. Ann. 2C:35-14 (West 2015)) 2016-19 -> (N.J. Stat. Ann. 2C:35-14 (West))
NY	2019	Cannot consider MOUD a violation of program rules	Diversion program	Under no circumstances shall a defendant who requires treatment for opioid abuse or dependence be deemed to have violated a release condition on the basis of his or her participation in medically prescribed drug treatments under the care of a health care professional licensed or certified under title eight of the education law, acting within his or her lawful scope of practice (§ 216.05) (N.Y. Crim. Proc. Law § 216.05 (McKinney))
	2019	Cannot restrict type of MOUD *(implied)*	Diversion program	No court shall require the use of any specified type or brand of drug during the course of medically prescribed drug treatments. (§ 216.05) (N.Y. Crim. Proc. Law § 216.05 (McKinney))
WA	2019	Cannot prohibit MOUD; cannot restrict type of MOUD	Treatment courts in regions that use certain types of funding	If a region or county uses criminal justice treatment account funds to support a therapeutic court, the therapeutic court must allow the use of all medications approved by the federal food and drug administration for the treatment of opioid use disorder as deemed medically appropriate for a participant by a medical professional. (Wash. Rev. Code Ann. § 71.24.580 (West 2019))

### Laws potentially facilitating MOUD access

Four states (Indiana, Mississippi, Missouri, and New Jersey) had a total of 12 laws requiring or permitting the court to take some action that could facilitate access to MOUD. Mississippi requires courts to conduct an assessment for appropriateness of MOUD, which could result in a court referring participants for MOUD. Missouri requires courts to make MOUD “available” and Mississippi requires courts to make available the “option” of MOUD. It is unclear whether the Mississippi law would require actual referrals to an MOUD provider or whether the law simply prohibits MOUD bans. Indiana permits courts to require MOUD, and New Jersey permits courts to require participation in rehabilitation programs, noting that such programs “may include” the use of MOUD. It is possible that the New Jersey law would permit a court to require MOUD. Table 2 provides the legal text and effective dates of the relevant laws in this category.

**Table 2:** Laws for facilitating MOUD access

State	Effective Years	Type of action	Applicability	Relevant legal text (citation)
IN	2015-2019	Permits courts to require MOUD	Problem-solving courts	A problem solving court may require an individual participating in a problem solving court to receive…medication assisted treatment, including a federal Food and Drug Administration approved long acting, nonaddictive medication for the treatment of opioid or alcohol dependence. (Ind. Code. Ann. § 33-23-16-24.5 (West))
MS	2019	Requires courts to assess for appropriateness of MOUD; Permits courts to refer participants to providers of “court-approved” MOUD	Criminal treatment courts	The court shall order the person to undergo an assessment that uses a standardized evidence-based instrument performed by a physician to determine whether the person has a diagnosis for alcohol and/or drug dependence and would likely benefit from a court-approved medication-assisted treatment indicated and approved for the treatment of alcohol and/or drug dependence by the United States Food and Drug Administration, as specified in the most recent Diagnostic and Statistical Manual of Mental Disorders published by the American Psychiatric Association. Upon considering the results of the assessment, the court may refer the person to a rehabilitative program that offers one or more forms of court-approved medications that are approved for the treatment of alcohol and/or drug dependence by the United States Food and Drug Administration (Miss. Code Ann. (§ 9-23-13 (West))
MS	2019	Requires making “court-approved” MOUD available in conformance with National Drug Court Institute guidelines	Treatment courts	All intervention courts shall make available the option for participants to use court-approved medication-assisted treatment while participating in the programs of the court in accordance with the recommendations of the National Drug Court Institute. (Miss. Code Ann. § 9-23-13 (West))
MO	2019	Requires completion of an SUD assessment by a physician; Requires making MOUD available	Drug courts or other diversion programs	Drug courts or other diversion programs that provide for alternatives to jail or prison for persons with a substance use disorder shall be required to ensure all persons under their care are assessed for substance use disorders using standard diagnostic criteria by a licensed physician who actively treats patients with substance use disorders. The court or other diversion program shall make available the MAT services covered under this section, consistent with a treatment plan developed by the physician. (RSMo § 191.1165 (West 2019))
NJ	2015-2019	Court may require a participant to use MOUD	Diversion program	As a condition of special probation, the court shall order the person to enter a residential treatment program at a facility licensed and approved by the Department of Human Services or a program of nonresidential treatment by a licensed and approved treatment provider, which program may include the use of medication-assisted treatment as defined in paragraph (7) of subsection f. (2C:35-14) 2015 -> (N.J. Stat. Ann. 2C:35-14 (West 2015)) 2016-19 -> (N.J. Stat. Ann. 2C:35-14 (West))

## Discussion

To our knowledge, this is the first systematic analysis of US state laws regarding MOUD use in problem-solving courts. We found that since 2005, nine states had a total of 37 relevant laws, with two overarching categories or themes: 1) laws that prohibit MOUD bans, and 2) laws that could facilitate MOUD access. Within these categories, we found significant variation in court actions prohibited or required. For example, facilitation laws ranged from requiring an assessment of MOUD appropriateness for court participants to allowing courts to mandate participants’ MOUD treatment. We also found some ambiguity in the meaning of state laws. For example, it is unclear whether a law requiring that courts make MOUD “available” means the court must refer participants to MOUD, must fund MOUD, or must take some other action.

Laws in our study are very recent, with only two of the 37 laws identified existing prior to 2015. The recency of laws found in our study could reflect a number of changed circumstances since 2015, including the following: increased policymaker and media attention to the opioid crisis, including the benefits of problem-solving courts and MOUD (Lopez, 2018; Shachar et al., 2019; McGinty et al., 2019; Gostin et al., 2017; Wickramatilake et al., 2017); successful legal cases arguing that MOUD prohibition in the criminal justice system is an Americans with Disabilities Act violation (Smith, 2019a; 2019b); widespread dissemination of best practice standards regarding MOUD by the National Association of Drug Court Professionals (National Association of Drug Court Professionals, 2015; National Association of Drug Court Professionals, 2018); increased availability of MOUD in carceral settings, including through state mandates to provide MOUD in prisons and jails (Thakrar et al., 2021); development of federal guidelines and technical assistance for implementation of MOUD in federally-funded justice settings (Use of Medication-Assisted Treatment for Opioid Use Disorder in Criminal Justice Settings, 2019); and increased educational opportunities about MOUD for court teams (Matusow et al., 2021; Andraka-Christou et al., 2020).

Importantly, some state laws explicitly forbid problem-solving courts from favoring one type of MOUD over another. Such laws may reflect growing awareness among policymakers that courts would otherwise favor naltrexone over agonist MOUD, even though agonist MOUD has a stronger evidence base for preventing overdose death (Wakeman et al., 2020; Morgan et al., 2019). For example, Missouri’s law says the court “shall not impose any limitations on the type of medication.” (MO 191.1165) Such language is important given that legal text merely saying a court must allow MOUD could lead a court to argue that it is complying with the law if it is allowing at least *some* type of MOUD (e.g., allowing naltrexone but not methadone). A qualitative study of Indiana courts and a survey of a sample of Florida courts found that some courts do prohibit one medication (typically methadone or buprenorphine) while allowing others (typically naltrexone) (Andraka-Christou et al., 2022; Andraka-Christou, 2017). Ironically, we identified an Indiana law that ostensibly allows judges to require participants to undergo any MOUD treatment, yet the text of the law highlights naltrexone, describing it as “nonaddictive” (IC 33–23–16-24.5) – incorrectly implying that methadone and buprenorphine are addictive.

While previous studies indicate that increased education is needed for court team members about MOUD, particularly to address biases and misconceptions (Matusow et al., 2013; Andraka-Christou & Atkins, 2020a; Andraka-Christou et al., 2019), state laws that operate regardless of court team member MOUD beliefs may be even more efficient at increasing court participant MOUD use. It is also possible that such state laws provide a powerful signal to court team members, including in other states, that MOUD is effective and is not “just substituting one addiction for another.” To our knowledge, however, no study has empirically assessed the effects of state laws regulating MOUD in courts.

Even though MOUD is the most effective OUD treatment, laws permitting courts to *require* MOUD treatment might be unethical, particularly if a participant is not interested in MOUD or if the type of MOUD the participant prefers is not locally available. Another concern is that laws requiring courts to permit MOUD may have no impact on participants if local providers are lacking. Relatedly, laws that hinge on the recommendation of a physician may not be practical when nurse practitioners and physician assistants have been legally permitted to prescribe buprenorphine since 2017 (Comprehensive Addiction and Recovery Act of 2016) and may be the only practitioners prescribing buprenorphine in very rural areas (Andrilla & Patterson, 2021). Furthermore, a Florida study found that most court team members perceived their court’s collaborating healthcare practitioner as *not* encouraging MOUD utilization (Andraka-Christou & Atkins, 2020b). Therefore, the practitioners from whom courts are most likely to seek recommendations about MOUD may be biased against MOUD.

While voluntary best practice guidelines do exist (National Association of Drug Court Professionals, 2015) and our study indicates an emerging trend toward greater lawmaking on this issue, model state legislation could help ensure consistency in MOUD policies across the nation. Such model legislation could include the following: prohibitions on courts using MOUD treatment to exclude people from program participation or program completion; prohibitions on court-imposed limitations to MOUD duration, medication type, and dose; requiring courts to permit MOUD anytime it is prescribed by a practitioner who is qualified to prescribe under federal and state law, including primary care physicians, nurse practitioners, and physician assistants; and requirements to connect people with OUD who are interested in MOUD to available MOUD providers.

Research is needed regarding the implementation of these laws, particularly since problem-solving courts have historically operated autonomously even within the same county. Research is also needed to examine potential effects of these laws on MOUD access, assessing whether problem-solving courts in states with laws prohibiting MOUD bans and/or facilitating access exhibit higher rates of court participant MOUD use. Lastly, future research should assess longer-term impacts of these laws on health outcomes of court participants.

Our study has several important limitations. Our data collection began with laws that explicitly used terms related to MOUD. It is possible that other state laws exist that encompass MOUD but use terminology not reflected in our search terms. For example, a law could exist requiring courts to make available the most effective treatments for SUD according to recent scientific studies, with such a law implying MOUD but not captured in our initial search. Also, our search only included laws from 2005 to 2019. While it is unlikely that many relevant laws existed prior to 2005, it is possible that new relevant laws have been passed since 2019. Finally, our study does not examine county-level and municipal policies related to MOUD use by problem-solving court participants – an important area for future research. Therefore, our finding that only nine states have statutes or regulations regarding MOUD access in problem-solving courts should be interpreted in the broader context of other policy mechanisms, including local laws, federal funding mechanisms, and voluntary practice guidelines, which could impact MOUD access in problem-solving courts even when state statutes/regulations on the subject are lacking.

## Conclusion

Poor MOUD access for problem-solving court participants is a known concern (Matusow et al., 2013; Andraka-Christou et al., 2019). Our study is the first to characterize the relative absence of state laws facilitating MOUD access and laws preventing MOUD bans in problem-solving courts, which suggests a general lack of state oversight regarding MOUD access for problem-solving court participants. Model state legislation could help ensure consistency in MOUD policies across the nation. Additionally, future research should explore implementation of these laws and effects on MOUD access and health outcomes of court participants.

## Supplementary Information

**Additional file 1.** Appendix A.

**Additional file 2.** Appendix B.

## Data Availability

No applicable. All state laws are in the public domain.
