# Supplementary material for: A national survey of state laws regarding medications for opioid use disorder in problem-solving courts
Source: Health Justice. 2022 Mar 31;10:14. doi: 10.1186/s40352-022-00178-6 (PMC8969254; doi:10.1186/s40352-022-00178-6)
Supplement: Supplementary file 1 — Additional file 1. Appendix A. [file 40352_2022_178_MOESM1_ESM.docx]

Appendix A:

Search terms utilized in Westlaw:

| “medication-assisted treatment” OR “medication assisted treatment” OR “medication-assisted treatments” OR “medication assisted treatments” OR (maintenance & opioid!) OR (maintain & opioid!) OR (maintenance & opiate!) OR (maintain & opiate!) OR buprenorphine OR (methadone! & maintain!) OR (methadone! & maintenance) OR (methadone! & addict!) OR (methadone & “use disorder”) OR (methadone & “use disorders”) OR naltrexone! OR “extended-release naltrexone” OR “extended release naltrexone” |
| --- |
