# Supplementary material for: A national survey of state laws regarding medications for opioid use disorder in problem-solving courts
Source: Health Justice. 2022 Mar 31;10:14. doi: 10.1186/s40352-022-00178-6 (PMC8969254; doi:10.1186/s40352-022-00178-6)
Supplement: Supplementary file 2 — Additional file 2. Appendix B. [file 40352_2022_178_MOESM2_ESM.docx]

**Identification of laws via Westlaw**

Laws identified from Westlaw (n = 14,951)

**Identification**

Laws screened

(n = 14,951)

Laws removed that were unrelated to SUD treatment

(n = 9,618)

Laws excluded that did not include at least one of the following terms: court, criminal, diversion program, or diversionary

(n = 4,448)

Laws uploaded into Dedoose software

(n = 5,333)

**Screening**

Laws excluded that did not pertain to problem-solving courts or only applied to non-court system actors

(n = 849)

Laws assessed for eligibility

(n = 885)

Laws included in review

(n = 36)

**Included**

*From:*  Page MJ, McKenzie JE, Bossuyt PM, Boutron I, Hoffmann TC, Mulrow CD, et al. The PRISMA 2020 statement: an updated guideline for reporting systematic reviews. BMJ 2021;372:n71. doi: 10.1136/bmj.n71

For more information, visit: <http://www.prisma-statement.org/>
